# High-Density Electromyography Provides New Insights into the Flexion Relaxation Phenomenon in Individuals with Low Back Pain

**DOI:** 10.1038/s41598-019-52434-9

**Published:** 2019-11-04

**Authors:** Carlos Murillo, Eduardo Martinez-Valdes, Nicola R. Heneghan, Bernard Liew, Alison Rushton, Andy Sanderson, Deborah Falla

**Affiliations:** Centre of Precision Rehabilitation for Spinal Pain (CPR Spine), School of Sport, Exercise and Rehabilitation Sciences, College of Life and Environmental Sciences, University of Birmingham, Birmingham, UK

**Keywords:** Rheumatic diseases, Skeletal muscle

## Abstract

Recent research using high-density electromyography (HDEMG) has provided a more precise understanding of the behaviour of the paraspinal muscles in people with low back pain (LBP); but so far, HDEMG has not been used to investigate the flexion relaxation phenomenon (FRP). To evaluate this, HDEMG signals were detected with grids of electrodes (13 × 5) placed bilaterally over the lumbar paraspinal muscles in individuals with and without LBP as they performed repetitions of full trunk flexion. The root mean square of the HDEMG signals was computed to generate the average normalized amplitude; and the spatial FRP onset was determined and expressed as percentage of trunk flexion. Smoothing spline analysis of variance models and the contrast cycle difference approach using the Bayesian interpretation were used to determine statistical inference. All pain-free controls and 64.3% of the individuals with LBP exhibited the FRP. Individuals with LBP and the FRP exhibited a delay of its onset compared to pain-free controls (significant mean difference of 13.3% of trunk flexion).  They also showed reduced normalized amplitude compared to those without the FRP, but still greater than pain-free controls (significant mean difference of 27.4% and 11.6% respectively). This study provides novel insights into changes in lumbar muscle behavior in individuals with LBP.

## Introduction

The flexion relaxation phenomenon (FRP) refers to the myoelectrical silence of the superficial paraspinal muscles which is observed during trunk flexion^1^. This phenomenon has been extensively reported in asymptomatic individuals but can be absent in individuals with low back pain (LBP)^1–3^. The FRP is examined by performing dynamic trunk flexion/extension, and the movement is then traditionally divided into trunk flexion, maximal voluntary flexion (MVF) (also known as the relaxation phase) and extension phases^4^.

Previously, studies have typically compared the FRP between individuals with and without LBP by quantifying the average electromyographic (EMG) amplitude during the MVF phase^5^. However, relying exclusively on the amplitude of muscle activity could lead to an incomplete interpretation of the alteration in muscle behaviour^4,6–8^. The addition of muscle activation timing (i.e. onset of the FRP) could provide a greater understanding of this phenomenon; however, its investigation has been limited to asymptomatic individuals^1–3,9,10^. To date, studies investigating the FRP of the paraspinal muscles have used classical bipolar EMG techniques^1–3,11,12^. It has been suggested however, that the multiple electrode locations (at different vertebral levels and distances from the spinous process) used to investigate the FRP of the superficial paraspinal muscles contribute in some extent to the large variability of findings across the literature (i.e. the proportion of individuals with LBP who do not exhibit FRP or the FRP onset timing)^1,2,4,13,14^. In contrast to classical approaches providing estimates of muscle activity from a single recording site, high-density EMG (HDEMG) provides a measure of muscle activity from multiple and closely spaced electrodes to generate a greater representation of muscle behaviour^15^ and to improve the reliability of amplitude estimates^16,17^. HDEMG has the additional advantage compared to bipolar EMG that it can be used to examine the spatial distribution of activity within the paraspinal muscles. Indeed, this method has revealed subtle variations of paraspinal muscle behaviour during sustained and dynamic contractions in individuals with and without LBP (e.g., less heterogeneous activation)^18–22^. Similarly, HDEMG could be used to study the spatial distribution of the timing of muscle activity^23^, particularly the FRP onset of the paraspinal muscles, but this has never been investigated.

The aim of the current study is to provide information and a better understanding of the muscle behavior of the superficial paraspinal muscles in individuals with LBP compared to pain-free controls. With that aim, we uniquely apply HDEMG to investigate paraspinal muscle activity during trunk flexion and quantify the spatial onset of the FRP.

## Results

### Sample characteristics

All pain-free controls displayed the FRP whereas 64.3% of individuals with LBP showed the FRP and 35.7% did not. The FRP was observed in all channels in all pain-free controls, as was the case for most individuals with LBP and the FRP, with the exception of two individuals who did not display the FRP in 29% and 33% of the channels of the HDEMG grid respectively.

The characteristics of the sample are presented in Table 1. The three groups were comparable in age, gender and BMI; and the LBP groups (with and without the FRP), did not differ in levels of pain, disability and kinesiophobia. There were no differences between individuals with LBP without the FRP, with FRP and pain-free controls in full flexion range of motion (94.27 ± 12.80, 91.50 ± 10.09 and 91.20 ± 12.42 respectively; *p* = 0.531) and in duration of the trunk flexion phase (3.56 ± 0.53 s, 3.66 ± 0.49 s and 3.85 ± 0.59 s respectively; *p* = 0.565). Since there were no differences between sides for all EMG variables in both the control group and LBP groups (p > 0.05), data were pooled between the left and right side.

**Table 1:** Baseline characteristics of the participants. LBP –Low Back Pain, BMI – Body Mass Index, NRS – Numeric Rating Scale for pain (0–4: mild pain, 4–6: moderate pain, 6–10: severe pain), ODI – Oswestry Disability Index (0% to 20%: mild disability, 21–40%: moderate disability, 41–60%: severe disability, 61–80%: crippled, 81–100%: symptoms exaggeration), TSK – Tampa Scale for Kinesiophobia.

Characteristic	LBP with FRP (n = 9) mean ± SD	LBP without FRP (n = 5) mean ± SD	Pain-free controls (n = 14) mean ± SD	p value
Age (years)	28.33 ± 11.01	28.2 ± 10.04	26.53 ± 4.90	*p* = 0.884
Sex (% male)	55.56	40	50	*p* = 0.856
BMI (Kg/m^2^)	25.46 ± 3.14	24.64 ± 3.80	23.35 ± 3.26	*p* = 0.597
NRS current pain (0–10)	2.22 ± 1.48	1.80 ± 1.64		*p* = 0.631
NRS usual pain (0–10)	3.22 ± 2.11	2.80 ± 1.92		*p* = 0.718
ODI (%)	11.44 ± 6.77	15.11 ± 5.75		*p* = 0.328
TSK (0–68)	34.4 ± 6.80	35.60 ± 6.03		*p* = 0.757

### FRP onset (%)

**Figure 1:**
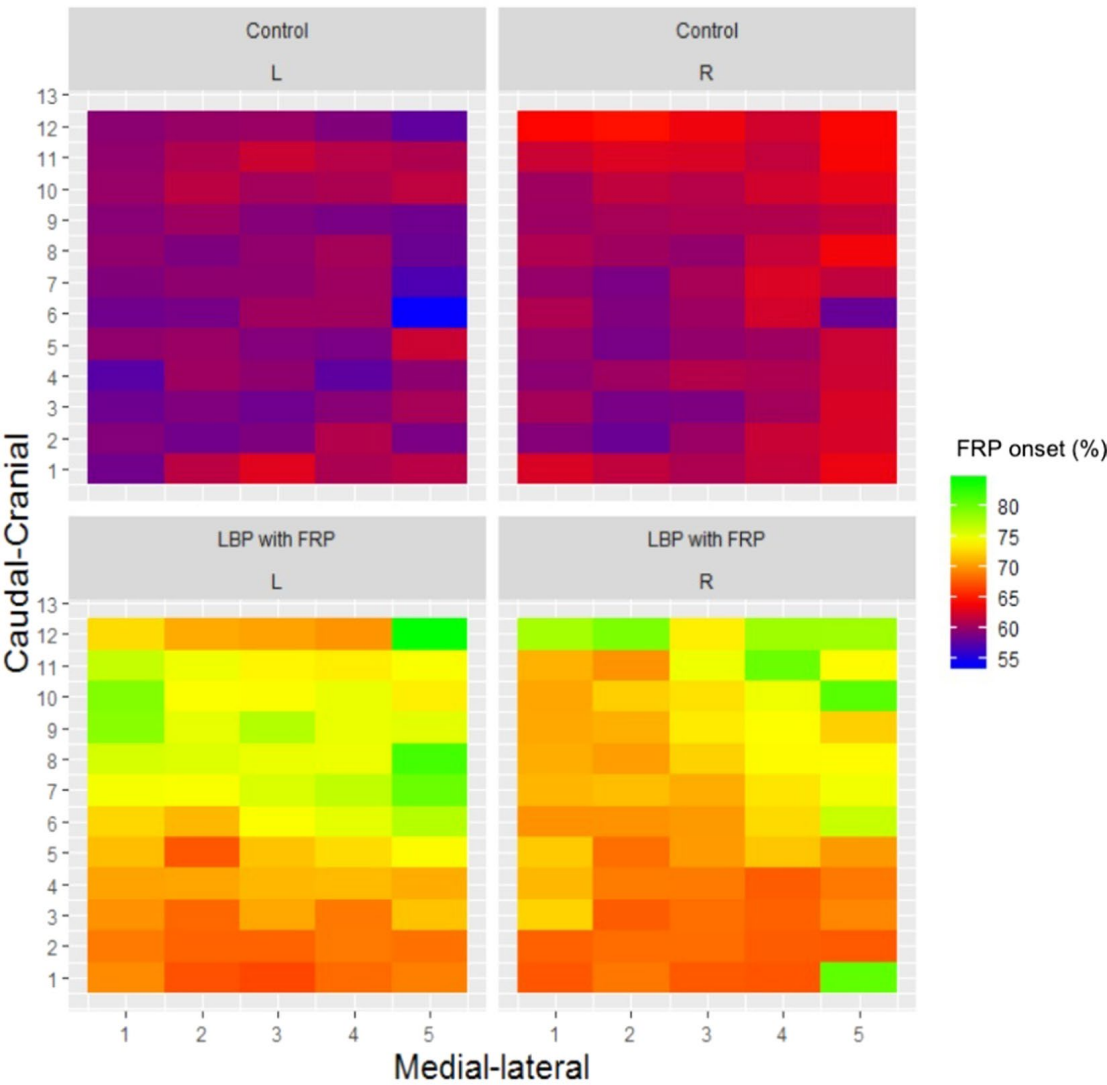
Group-averaged spatial map of the FRP onset (%) of the lumbar paraspinal (both sides) muscle activity relative to the phase of trunk flexion. Abbreviations: R = right, L = left, LBP = low back pain, FRP = flexion relaxation phenomenon.

**Figure 2:**
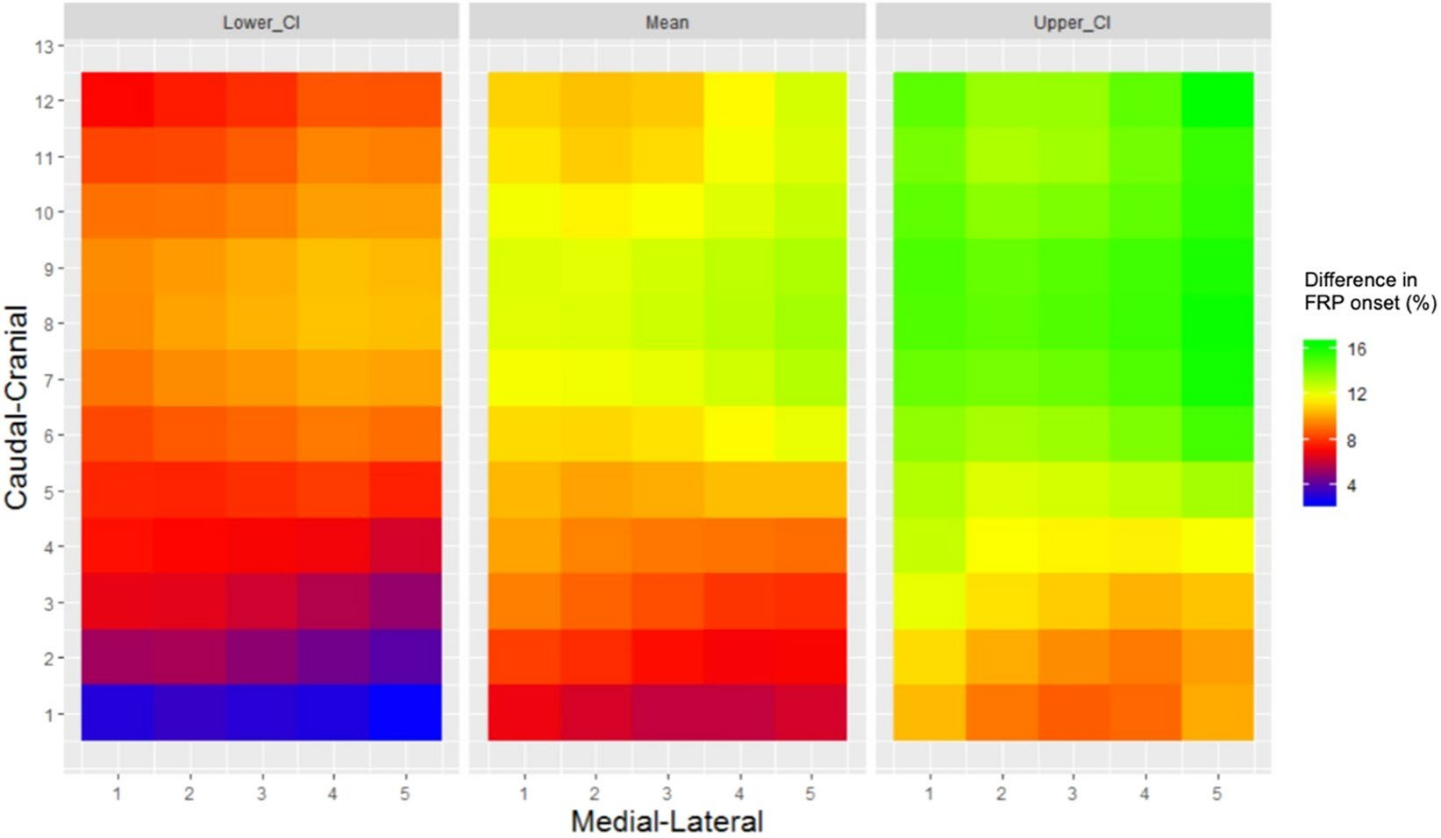
Mean effects with 95% Bayesian Confidence Interval (CI) of the difference between groups of theFRP onset (%) of the lumbar paraspinal muscle activity relative to the phase of trunk flexion. Abbreviations: FRP = flexion relaxation phenomenon.

The average onset of the FRP relative to the stage of trunk flexion is presented in Fig. 1. The SS-ANOVA model fit was R^2^ = 15.6%. As demonstrated in Fig. 1, the individuals with LBP who showed the FRP, demonstrated a later onset of the FRP relative to the pain-free controls. Figure 2 illustrates the mean effects of the difference between groups for the FRP onset (%) of lumbar paraspinal muscle activity relative to the stage of trunk flexion. The mean delay between groups was 13.3% (95% CI; 10.5 to 16.2). As demonstrated in Fig. 2, the spatial distribution of the FRP onset was heterogeneous, with an indication that the greatest difference between groups was present in more cranial regions of the lumbar paraspinal muscles.

### EMG amplitude during trunk flexion and MVF phases

**Figure 3:**
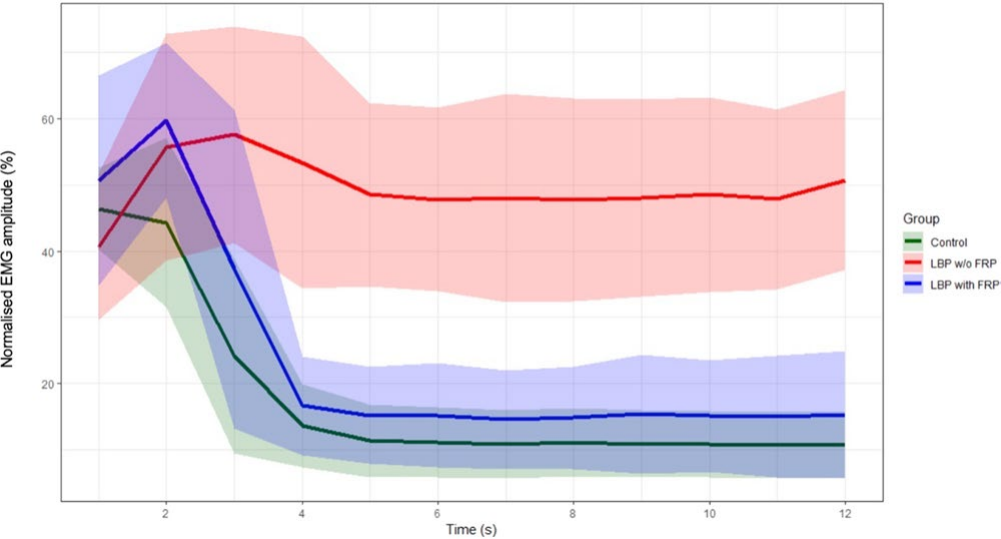
Group-averaged % EMG amplitude and standard deviation (SD) for each group, across the duration of the flexion task. Abbreviations: w/o = without, LBP = low back pain, FRP = flexion relaxation phenomenon.

**Figure 4:**
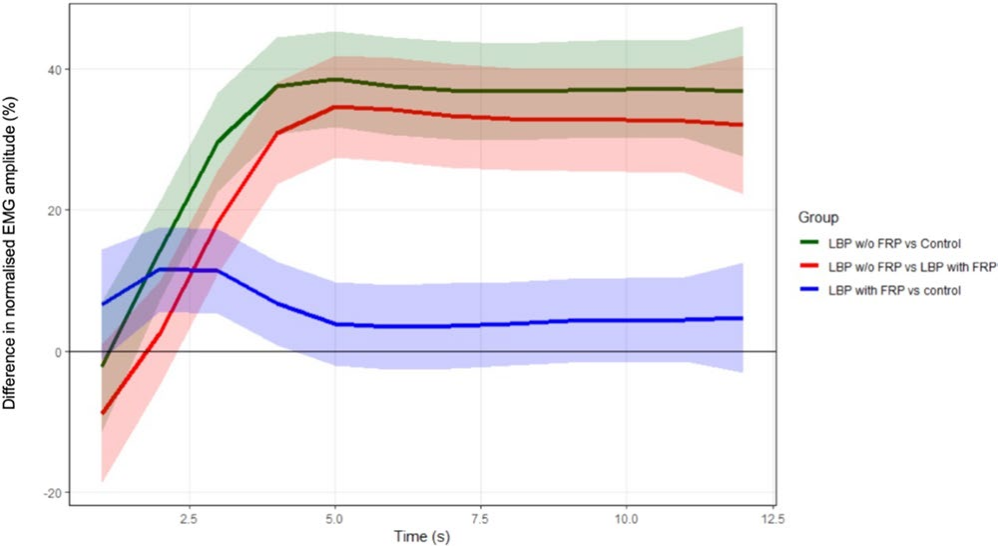
Mean effects with 95% Bayesian Confidence Interval (CI) of each pair-wise group difference of % EMG amplitude, across the duration of the flexion task. Abbreviations: LBP = low back pain, FRP = flexion relaxation phenomenon.

The group averages for the normalised EMG amplitude (%) during trunk flexion and MVF phases are presented in Fig. 3. The SS-ANOVA model fit was R^2^ = 74.7%. The largest between-group difference in EMG amplitude was observed between individuals with LBP without the FRP compared to pain-free controls (mean difference of 38.5% [95% CI; 31.7 to 45.3] and this peaked at 5 s (beginning of MVF phase) (Fig. 4). Individuals with LBP who exhibit the FRP also had 27.4% (95% CI; 27.4 to 41.9) greater EMG amplitude across the task than those without the FRP, which similarly peaked at 5 s into the trunk flexion task (Fig. 4). There was also a significant difference between individuals with LBP and with FRP compared to pain-free controls (mean difference of 11.6% [95% CI; 5.6 to 17.6]), with the greatest difference in EMG amplitude observed at 2 s (first half of the trunk flexion phase) (Fig. 4).

## Discussion

This study provides new insights into the lumbar muscle behavior during trunk flexion movements between individuals with and without LBP and supports the notion that individuals with LBP display heterogeneous changes in muscle behaviour. The large variability of results reported for the FRP in individuals with LBP is likely to be partially attributed to the variation of the electrode location over the paraspinal muscles in previous bipolar EMG studies^4^. Thus, the use of HDEMG in this study allowed for a more comprehensive estimation of the FRP of the superficial paraspinal muscles and a better understanding of how this differs between individuals with and without pain. So far, HDEMG research has been focused on examining how the level of muscular activity spatially distributes (shift of EMG amplitude) across the paraspinal muscles during endurance tasks^18–22^. The application of smoothing spline Analysis of Variance models (SS-ANOVA) models and the contrast cycle difference (CCD) approach using the Bayesian interpretation proposed in the current study permitted to spatially evaluate the FRP onset and investigate how this is regionally redistributed in individuals with LBP who exhibit it. Although some individuals with LBP displayed the FRP, this study uniquely shows that its onset is delayed compared with pain-free controls.

On the other hand, the conclusions on the FRP alterations in individuals with LBP have been traditionally determined from an analysis of the mean EMG amplitude recorded during the entire phase, either trunk flexion or MVF^5,24^. Although higher muscular activity during the MVF phase have commonly been observed in individuals with LBP compared to pain-free controls following this approach, conflicting findings have been reported for the trunk flexion phase^5,24^. This study proposes a new innovative statistical approach which allowed for the analysis of the muscle activity amplitude throughout flexion and at MVF phases, providing a comprehensive comparison between individuals with LBP with and without FRP compared to pain-free controls. This revealed more subtle variations in paraspinal muscle behaviour and showed its advantages when compared to the traditional approach of averaging the EMG amplitude over the entire phase^4,5^. During the first half of trunk flexion, individuals with LBP who displayed FRP exhibited a similar increase in EMG amplitude to those individuals with LBP and without FRP; but similar rate of relaxation to the pain-free controls was observed from the second half of the movement. On the contrary, this was not observed for those with LBP who did not display the FRP. Additionally, as expected, individuals with LBP who displayed the FRP showed lower activity of their paraspinal muscles during MVF compared to those with an absent FRP; but this was still slightly higher than pain free controls. Taken together, this suggests that individuals with LBP showed an alteration of the FRP to some extent and that the presence/absence of the FRP should not be considered as a dichotomy. It should be noted however, that the individuals with LBP included in the present study had fairly low levels of pain and disability and thus further research should investigate whether similar findings exist in individuals with more severe symptoms or levels of disability^25,26^.

### FRP onset during trunk flexion

The percentage of individuals with LBP who did not exhibit the FRP in the present study is in line with previous reports by Floyd and Silver^1^ and Triano and Schultz^2^ (32% and 43% respectively). Although some individuals with LBP did display the FRP, the onset was delayed relative to pain-free controls. This was typically identified in every channel of the HDEMG grid, suggesting that the entire superficial paraspinal musculature within the lumbar region was affected. Thus, not only is the FRP absent in many individuals with LBP, possibly as a protective or a reactive response to pain, but in those that do display the FRP the response is delayed. The delay was more evident in the cranial region of the electrode, which is the region that covers the longissimus muscle; in contrast to the caudal region which likely covered more of the superficial multifidus^27^. Interestingly, Schinkel-Ivy, *et al*.^14^ reported an earlier FRP onset of the longissimus compared to the superficial multifidus in pain-free controls; suggesting that the multifidus remains activate longer during trunk flexion, possibly for postural support. It has been hypothesised that the absence of the FRP in individuals with LBP is due to failure of the interplay of lumbar structures, that is, relaxation of the superficial paraspinal muscles does not occur in order to compensate for inefficient or injured capsuloligamentous structures^4^. Our findings agree with this hypothesis of compensation in the lumbar spine; but in this case, within the paraspinal muscles. The spatial redistribution of the FRP onset observed in individuals with LBP, where the longissimus muscle remains active longer, may reflect a redistribution of activity in order to provide further support of the lumbar region. Interestingly, in line with current findings, a delay of the FRP onset of the longissimus has been observed in pain-free individuals after the addition of loads or after fatiguing tasks^9,28,29^. Thus, taken together these findings suggest that in presence of pain or fatigue, the paraspinal muscles exhibit a redistribution of activity in order to meet the movement demands and control the spine during trunk flexion. However, this remains speculative and further research must investigate this hypothesis.

### Amplitude of paraspinal muscle activity during trunk flexion and at MVF

FRP research has consistently found higher muscular activity in individuals with LBP compared to pain-free controls during the MVF phase^5^. However, findings are conflicting in terms of the trunk flexion phase with some studies reporting higher muscular activity in individuals with LBP compared to pain-free controls and others showing lower muscular activity or no differences^5,24^. Traditionally, the evaluation of the FRP has been traditionally limited to comparison of the amplitude of muscle activity at the MVF phase of individuals with LBP as a whole to pain-free individuals^5^. However, this may lead to a washout effect where findings of a proportion of individuals with LBP who display FRP are concealed with the results from the others who do not^30^. This study revealed significant differences in levels of muscle activity during the trunk flexion and MVF phases between individuals with LBP with and without FRP supporting the concept of washout effect.

The statistical approach proposed in the current study clearly showed two stages in the pain-free controls during trunk flexion; a slight decrease in EMG amplitude from the start to approximately halfway through trunk flexion (0–2 s) followed by a more rapid decline of EMG (relaxation rate) (Fig. 4). On the contrary, the EMG amplitude increases in a similar manner in both LBP groups during the first half of trunk flexion. In the second half of trunk flexion, whereas a similar relaxation rate of the EMG amplitude to pain-free controls is observed for the individuals with LBP and with FRP; those with LBP who did not display FRP maintain a similar level of EMG amplitude following the initial increase.

The current findings revealed that those individuals with LBP who display the FRP still showed slightly higher EMG amplitude than pain-free individuals at the MVF phase. Also, as expected, higher EMG amplitude was observed in individuals with LBP and an absent FRP compared with individuals with FRP; both pain-free controls and those with LBP. Interestingly, it is also evident from Fig. 4 that the level of paraspinal muscle activity did not vary throughout the MVF in any group; and the individuals with LBP who showed the FRP did not reach relaxation levels comparable to those found in pain-free controls by the end of the MVF. These findings are in agreement with Dankaerts, *et al*.^30^, who identified a subgroup of individuals with LBP who displayed higher muscular activity compared to pain-free controls and other individuals with LBP. Similarly, in a recent study which classified individuals with LBP based on trunk kinematics, the majority of the pain-free controls were included in a subgroup with the lowest levels of muscular activity during MVF, whereas individuals with LBP were heterogeneously distributed into subgroups with increasing muscular activity^31^. Factors such as the degree of disability or level of kinesiophobia have been used to explain the heterogeneity of muscle activity during MVF in individuals with LBP; however, evidence is inconclusive^32,33^. The recent findings reported by Laird, *et al*.^34^ together with previous findings reported by Dankaerts, *et al*.^30^ appears to suggest that this heterogeneity could be associated with differing trunk kinematics, but this should be further explored in future research.

In conclusion, the current findings overall suggest that individuals with LBP display heightened activation of the superficial paraspinal muscles, which results in either an absent or delayed FRP, and increased muscle activity during the MVF to some extent. This may be indicative of a protective strategy to avoid pain but further research is needed to investigate the consequences of this strategy to the persistence of LBP^35^.

## Methods

Fourteen participants with non-specific LBP and 14 pain-free controls aged between 20 to 55 years-old were recruited from the staff and student community of the University of Birmingham, United Kingdom. The study was conducted at the Centre of Precision Rehabilitation for Spinal Pain (CPR Spine) at the University of Birmingham. The LBP group included participants who reported continuous LBP for more than 3 months or non-continuous LBP for more than 6 months with pain reported on at least half of the days^36^. The pain-free group included participants without any history of LBP. Exclusion criteria for both groups included neurological or respiratory disorders, scoliosis, pregnancy or previous spinal surgery. A further exclusion for the LBP group was receiving treatment from a health care professional at the time of recruitment. Pain intensity (on the day of the laboratory session and usual pain during the previous week), disability and kinesiophobia were evaluated in those with LBP with the Numerical Rating Scale (NRS)^37^, the Oswestry Disability Index (ODI)^26^ and the Tampa Scale for Kinesiophobia (TSK)^38^ respectively. Ethical approval for the study was obtained from the University of Birmingham Ethics Committee (ERN_17–0782) and the procedures were conducted according to the Declaration of Helsinki. Written informed consent was obtained from all participants prior to the study, and the rights of the subjects were protected. This study is reported according to the STROBE checklist^39^.

### Procedure

To minimise inter-subject variability, all participants were positioned with their feet shoulder width apart and with their hallux placed in contact with a horizontal line on the floor. The participants were requested to perform three non-consecutive repetitions of trunk flexion returning to the starting position in standing to assess the FRP^4^. A 60-second break was provided between each repetition. Participants were instructed not to bend their knees during the flexion movement and reach a relaxed full flexion position. The examiner demonstrated the task until the participant was satisfied that they understood it and a single practice repetition was performed to familiarise the participant with the task. The speed of trunk flexion was standardised with a metronome set at 60 beats per minute and each repetition was divided into 3 phases; trunk flexion (4 s), full flexion position (8 s) and return to neutral (4 s).

### Electromyography

HDEMG was acquired from the superficial lumbar paraspinal muscles bilaterally using two 13 × 5 semi-disposable 2D electrode grids (OT Bioelettronica, Torino, Italy). Each grid consisted of 13 rows and 5 columns of electrodes (1-mm diameter, 8-mm interelectrode distance), with one electrode absent from one corner in each grid to provide a directional reference. The participant’s skin in the area which electrodes would be adhered to was prepared by gentle local abrasion using an abrasive paste (Nuprep skin prep gel, Weaver and Company, USA) and cleaned with water following recommendations^40^. An adhesive foam was applied to the surface of the electrode grid and the holes representing the location of the electrodes were filled with electro-conductive paste (SPES Medica, Italy). The two electrode grids were then positioned over the superficial paraspinal muscles with the lower edge of the grid 2 cm lateral to the lumbar spinous processes at the level of L5, extending to approximately L2, as previously described by Falla, *et al*.^20^ and Martinez-Valdes, *et al*.^21^. Reference electrodes were placed on the spinous process of the vertebrae prominent and over the right anterior superior iliac crest. Hypoallergenic flexible tape was used to ensure good skin adherence of the electrode grids during the task. This tape was attached with the trunk in mid-flexion so as not to not limit flexion movement.

Lumbar flexion movement was quantified using a twin axis electrogoniometer (SG 150B, Biometrics Ltd., UK), which is a reliable and valid tool to evaluate lumbar spinalmovement^41^. The lower sensor of the electrogoniometer was placed at the centre of the iliac crest in line with the greater trochanter and the upper sensor was positioned over the right side of the participant’s trunk corresponding approximately with the level of T12. The electrogoniometer was calibrated to 0° with the participant in standing. Both EMG signals and angular data were sampled at 2048 Hz and amplified (Quattrocento, OT Bioelettronica, Torino, Italy; -3dB, bandwidth 10–500 Hz) by a factor of 150 and converted to digital form by a 16-bit analogue-to-digital converter.

### Data processing

Signal quality was monitored during the performance of the task and off-line at the time of the analysis. Those channels with poor signal quality (low signal to noise ratio) due to movement artefacts or poor skin-electrode contact were excluded as described previously by Falla, *et al*.^20^ and Testa, *et al*.^42^. The 64 monopolar signals from each electrode grid were filtered using a band-pass filter with corner frequencies between 20–350 Hz, and processed to produce 59 bipolar EMG signals^20,21^. The root mean square (RMS) value was obtained by computing the RMS from each bipolar signal, from adjacent, non-overlapping epochs in windows of 1 s for trunk flexion (4 epochs) and MVF (8 epochs). The bipolar signals were then averaged on each epoch, to produce a mean RMS value per electrode grid. The EMG RMS amplitude values of each second were normalised to the peak value during the extension phase^31^.

**Figure 5:**
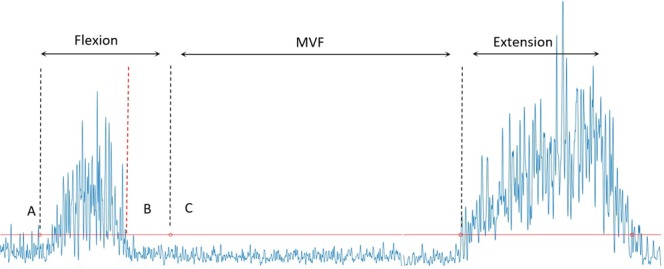
FRP onset determination on the EMG rectified signal of one channel of the HDEMG grid. The red horizontal line represents the three standard deviations of mean of baseline EMG activity measured during quiet standing. The red vertical line (**B**) denotes the FRP onset in relation to the start (**A**) and the end (**B**) of the truck flexion determined by the electrogoniometer.

To determine the onset of the FRP (offset of EMG activity), the EMG data was full wave rectified and low-pass filtered at 50 Hz. The EMG offset was identified with a semiautomatic method^43^, where EMG signals were visually inspected as previously described^44^. The FRP onset was defined as the point where the EMG signal amplitude decreased below three standard deviations of mean of baseline EMG activity measured during quiet standing^44^ (Fig. 5). The EMG offset value was detected for the included channels on each grid and these channels were then used to generate a topographical map of the FRP onset. We defined that the FRP was present if the EMG offset was observed during trunk flexion in >50% of the channels of the HDEMG electrode grid. In this case, those channels with an absent FRP would be excluded for the analysis of the FRP onset map. For the analysis, the mean of the three repetitions was calculated to obtain a single representative value for each EMG variable (amplitude and FRP onset). Since the duration of the trunk flexion phase was not different between pain-free controls and individuals with LBP who displayed FRP (see results below), the data could be directly compared between groups. The point where the FRP onset occurred within the trunk flexion phase was calculated and expressed as a percentage in order to allow between-subject comparison^10^.

### Statistical inference

Descriptive statistics (mean, standard deviation [SD]) were used to analyse demographic data with inferential analysis, including parametric and non-parametric tests to compare groups. Full flexion range of motion and the duration of the trunk flexion phase (seconds) was compared between the 3 groups to investigate if the differences in FRP onset between groups were due to differences in full flexion range of motion and/or performance time between groups and confirm adherence to the timing imposed during the task. The two dependent variables were (A) a two-dimensional (2D) signal of normalised FRP onset (5 × 12) and, (B) a one-dimensional (1D) signal of normalised EMG amplitude (RMS) averaged across all channels within the electrode grid (12 × 1). The independent variables were group (LBP with FRP, LBP without FRP, and pain-free controls).

To quantify between group differences for the dependent variables, mixed-effects SS-ANOVA were used^45–47^. All analyses were carried out using the bigssa function in bigsplines package^48^ in the R environment^49^. All codes are included in the Supplementary Material.

For the dependent variable of FRP onset, a cubic thin-plate smoothing spline was used for the independent variable “space”, whilst group (LBP with FRP and pain-free controls) and side (right vs left) were treated as nominal variables. For the dependent variable of EMG amplitude, a cubic smoothing spline was used for the independent variable “time”, whilst group LBP with FRP, LBP without FRP, and pain-free controls) was treated as a nominal variable. The generalize cross-validation method^50^ was used to estimate the model’s smoothing parameters^51^. The CCD approach using the Bayesian interpretation of a smoothing spline was used for statistical inference^47,52^. Specifically, significance was defined when the 95% CI of the mean pairwise group CCD does not contain zero^47,52^. For the dependent variable of FRP onset, a single pairwise CCD was computed between LBP with FRP vs control. For the dependent variable of EMG amplitude, three pairwise CCD were computed (LBP with FRP vs LBP without FRP, LBP with FRP vs control, LBP without FRP vs control).

## Supplementary information

Code for EMG timing (FRP onset) analysis

## Data Availability

All codes used for the statistical inference are available as a Supplementary File. The datasets analyzed during the current study are available from the corresponding author on reasonable request.
