# Supplementary material for: High-Density Electromyography Provides New Insights into the Flexion Relaxation Phenomenon in Individuals with Low Back Pain
Source: Sci Rep. 2019 Nov 4;9:15938. doi: 10.1038/s41598-019-52434-9 (PMC6828973; doi:10.1038/s41598-019-52434-9)
Supplement: Supplementary file 1 — Code for EMG timing (FRP onset) analysis [file 41598_2019_52434_MOESM1_ESM.pdf]

# HIGH-DENSITY ELECTROMYOGRAPHY PROVIDES NEW INSIGHTS INTO THE FLEXION RELAXATION PHENOMENON IN INDIVIDUALS WITH LOW BACK PAIN

Carlos Murillo[1], Eduardo Martinez-Valdes[1], Nicola R Heneghan[1], Bernard Liew, [1] Alison Rushton[1], Andy Sanderson, [1] Deborah Falla[1]*

[1]Centre of Precision Rehabilitation for Spinal Pain (CPR Spine)
School of Sport, Exercise and Rehabilitation Sciences, College of Life and Environmental Sciences, University of Birmingham, UK

This study was approved by the University of Birmingham ethics committee and the procedures were conducted in agreement with the Declaration of Helsinki (ERN_17-0782).

Corresponding Author – Deborah Falla
d.falla@bham.ac.uk

**Code for EMG timing (FRP onset) analysis**

````
# Create merged data in long format
```{r}
modify = function (df) {
  df %>%
  gather(c (5:9), key = COL, value = EMG) %>%
    mutate (ROW = gsub ("R", "",.$ROW),
            ROW = as.numeric (ROW),
            COL = gsub ("C", "",.$COL),
            COL = as.numeric (COL)) %>%
    arrange (TIME, SIDE, REP, ROW, COL)
}

data.long = lapply (SheetList, modify)
df.long = bind_rows(data.long, .id = "ID") %>%
  mutate (ID = as.factor(ID),
          SIDE = as.factor (SIDE)) %>%
  mutate (GRP = ifelse (grepl("PH",.$ID), "CON", "LBP"),
          GRP = as.factor (GRP)) %>%
  mutate (EMG = ifelse (EMG > 100, NA,
                        ifelse (EMG < 0, 0, EMG)))

```
# Create two LBP groups, LBP with relaxation and without relaxation
```{r}

df.long = df.long %>%
  mutate (GRP2 = GRP,
          GRP2 = ifelse (GRP2 == "CON", "CON",
                         ifelse (ID %in% c("PP2", "PP5", "PP6", "PP8", "PP9") &
                                   TIME == "OFF","LBP_NO", "LBP_YES")),
          GRP2 = as.factor (GRP2))

```
# Plots
## Group plots for offset
```{r fig.height=10}
df.plot = df.long %>%
  filter (TIME == "OFF")%>%
  group_by(GRP2, TIME, SIDE, ROW, COL) %>%
  summarize (timing = mean(EMG, na.rm = T)) %>%
  ungroup() %>%
  mutate (GRP2 = fct_recode(GRP2,
                            "Control" = "CON",
                            "LBP w/o FRP" = "LBP_NO",
                            "LBP with FRP" = "LBP_YES")) %>%
````
````

````
  filter (GRP2 != "LBP w/o FRP")

data_range <- (range(df.plot$timing, na.rm = T) - min(range(df.plot$timing, na.rm = T))) /
diff(range(range(df.plot$timing, na.rm = T)))
vals <- seq(data_range [[1]], data_range [[2]], length.out=100)

my_palette <- colorRampPalette(c("blue", "red", "yellow", "green"))

ggplot(df.plot  , aes( COL, ROW)) +
  geom_tile(aes(fill = timing)) +
  facet_wrap( GRP2 ~ SIDE, ncol = 2) +
  scale_fill_gradientn(colours = my_palette (100), values = vals, name = "% FRP Onset") +
  ggtitle ("Figure") +
  ylab ("Caudal-Cranial") +
  xlab ("Medial-lateral") +
  theme (axis.title.x = element_text (size = 16),
         axis.title.y = element_text(size = 16)) +
  scale_y_continuous(breaks=seq(1,13,1))

```
# BigSSA
```{r}
dat_off = df.long %>%
  filter (TIME == "OFF") %>%
  filter (GRP2 != "LBP_NO") %>%
  mutate (GRP2 = factor(GRP2, levels = c("CON", "LBP_YES"))) %>%
  na.omit

side.knots <- c( "L","R")
row.knots = seq (1:12)
col.knots <- seq (1:5)
grp.knots <- c("CON", "LBP_YES")
knotID <- NULL
for (ee in side.knots) {
  for(ii in row.knots){
    for(jj in col.knots){
      for(kk in grp.knots){
        ix <- which(dat_off$SIDE == ee & dat_off$ROW == ii & dat_off$COL == jj &
dat_off$GRP2== kk)
        knotID <- c(knotID, ix[1])
      }
    }
  }
}

dat_off$chanmat = as.matrix (cbind(dat_off$ROW,dat_off$COL))
````

~~~~
off_ssmod_1 =bigssa (EMG ~chanmat*GRP2*SIDE,
        type=list(chanmat="tps",GRP2 ="nom", SIDE = "nom"),
        rparm=list(chanmat =0.01,GRP2 =1,SIDE=1),
        nknots=knotID,
        data = dat_off,
        skip.iter=T)
off_ssmod_1$info
```
## Get CCD for paired conditions
```{r}

iname = c("chanmat", "GRP2", "chanmat:GRP2")

row = seq (1:12)
col <- seq (1:5)
grp <- c("CON","LBP_YES")

newdata =  expand.grid(ROW=row, COL = col, GRP2=grp)
newdata$chanmat = as.matrix (cbind(newdata $ROW,newdata $COL))

yhat = predict(off_ssmod_1, newdata = newdata, include = iname, includeint = T,  se.fit=T,
design = T)

ccd_fit = newdata
ccd_fit$emg = yhat$fit
ccd_fit$X = yhat$X

con = ccd_fit[ccd_fit$GRP2 == "CON",]
lbp_y = ccd_fit[ccd_fit$GRP2 == "LBP_YES",]

colnames(con)[c(5,6)] = c("emg_con", "X_con")
colnames(lbp_y)[c(5,6)] = c("emg_lbp_y", "X_lbp_y")

ccd = lbp_y[c(5,6)]- con[c(5,6)]

const = con[,1:3]
ccd2 = cbind (const, ccd[,1] )
ccd2$X = as.matrix (ccd[,-1])

colnames (ccd2)[c(4:5)] = c("timing_diff", "X")
coefsqrt = off_ssmod_1 $modelspec$coef.csqrt

### get Bayesian standard errors for CCDs
ccd2$se = sqrt(rowSums(((ccd2$X) %*% coefsqrt)^2))

```
## Plot of mean difference between LBPY minus Con
~~~~

```{r}
################################## Plots for LBP_Y minus
Con####################################
level = 0.95
cval = qnorm(1 - (1 - level)/2)

ccd.plot = ccd2 %>%
  select (-X) %>%
  mutate (yhat = timing_diff,
          upper =  timing_diff + cval*se,
          lower =  timing_diff - cval*se) %>%
  mutate (sig = ifelse (.$lower > 0 | .$upper < 0, "s", "ns"))

data_range <- (range(ccd.plot$yhat) - min(range(ccd.plot$yhat))) /
diff(range(range(ccd.plot$yhat)))
vals <- seq(data_range [[1]], data_range [[2]], length.out=100)

my_palette <- colorRampPalette(c("blue", "red", "yellow", "green"))

ggplot(ccd.plot , aes(COL,ROW)) +
  geom_tile(data = ccd.plot %>% filter (sig == "s"), aes(fill = yhat)) +
  scale_fill_gradientn(colours = my_palette (100), values = vals) +
  geom_tile(data = ccd.plot %>% filter (sig == "ns"), fill = "white") +
  labs(fill="% Timing") +
  ylab ("Caudal-Cranial") +
  xlab ("Medial-Lateral") +
  ggtitle ("Figure . Significant mean differences")

```
## Plot of mean difference wiht CI between LBPY minus Con
```{r}
level = 0.95
cval = qnorm(1 - (1 - level)/2)

ccd.plot = ccd2 %>%
  select (-X) %>%
  mutate (Mean = timing_diff,
          Upper_CI =  timing_diff + cval*se,
          Lower_CI  =  timing_diff - cval*se) %>%
  mutate (sig = ifelse (.$Lower_CI > 0 | .$Upper_CI < 0, "s", "ns"))  %>%
  gather (c(6:8), key = effects, value = EMG) %>%
  mutate (effects = factor (effects, levels = c("Lower_CI", "Mean", "Upper_CI")))

data_range <- (range(ccd.plot$EMG) - min(range(ccd.plot$EMG))) /
diff(range(range(ccd.plot$EMG)))
vals <- seq(data_range [[1]], data_range [[2]], length.out=100)
```

```
ggplot(ccd.plot , aes(COL,ROW)) +
  geom_tile( aes(fill = EMG)) +
  scale_fill_gradientn(colours = my_palette (100), values = vals) +
  facet_wrap(~effects, ncol = 3) +
```

**Code for EMG amplitude analysis**

```
# Import data

```{r}
EMG_RMS <- read_delim("EMG_RMSv3.txt", "\t",
escape_double = FALSE, col_types = cols(GRP = col_factor(levels = c("CON",
"LBP"))), trim_ws = TRUE)

Demograp <- read_delim("Demograp.txt", "\t",
                    escape_double = FALSE, col_types = cols(GRP = col_factor(levels = c("CON",
                    "LBP")), SEX = col_factor(levels = c("1", "2"))), trim_ws = TRUE)
df = EMG_RMS %>%
  inner_join(Demograp, by = c("ID", "GRP")) %>%
  as.data.frame ()


```


# Plot some data
```{r fig.height=5, fig.width=5, message=FALSE, warning=FALSE}
df.plot =   df %>%
  gather (c(3:14), key = TIME, value = EMG) %>%
  mutate (TIME = as.numeric (TIME))%>%
  arrange (ID, TIME) %>%
  mutate (GRP2 = GRP,
        GRP2 = ifelse (GRP2 == "CON", "CON",
                    ifelse (ID %in% c("PP2", "PP5", "PP6", "PP8", "PP9"),"LBP_NO",
"LBP_YES")),
        GRP2 = as.factor (GRP2)) %>%
  group_by(GRP2, TIME) %>%
  summarize (EMGmean = mean (EMG),
          EMGsd = sd (EMG))

ggplot (df.plot) +
  geom_line(aes (x= TIME, y = EMGmean,  colour = GRP2), size = 1.5) +
  geom_ribbon(aes(x= TIME, ymin = EMGmean - EMGsd, ymax = EMGmean + EMGsd,  fill =
GRP2), alpha = 0.2) +
  scale_colour_manual(values = c("darkgreen", "red", "blue"),
                  name = "Group",
                  labels = c("Control", "LBP w/o FRP", "LBP with FRP")) +
```
```

~~~
  scale_fill_manual(values = c("darkgreen", "red", "blue"),
                    name = "Group",
                    labels = c("Control", "LBP w/o FRP", "LBP with FRP")) +
  scale_alpha_discrete(range = seq (0.9,1.0,length.out = 28)) +
  guides(alpha =FALSE) +
  labs (
    title = "Figure",
    x = "Time (s)",
    y = "% EMG amplitude"
  ) +
  theme (axis.title.x = element_text (size = 16),
         axis.title.y = element_text(size = 16),
         legend.text=element_text(size=16),
         legend.title=element_text(size=16)) +
  scale_x_continuous(breaks=c(2,4,6,8,10,12)) +
  theme_bw()


```



# Prepare the data
```{r}
dat = df %>%
  gather (c(3:14), key = TIME, value = EMG)  %>%
  mutate (TIME = as.numeric (TIME),
          EMG = EMG) %>%
  arrange (ID, TIME) %>%
  mutate (GRP2 = GRP,
          GRP2 = ifelse (GRP2 == "CON", "CON",
                         ifelse (ID %in% c("PP2", "PP5", "PP6", "PP8", "PP9"),"LBP_NO",
"LBP_YES")),
          GRP2 = as.factor (GRP2))
dat$TIME = as.numeric (dat$TIME)
```

# BigSSA
```{r}
time.knots = unique(dat$TIME)
grp.knots <- c("CON","LBP_NO", "LBP_YES")
knotID <- NULL

    for(jj in time.knots){
      for(kk in grp.knots){
        ix <- which(dat$TIME == jj & dat$GRP2== kk)
        knotID <- c(knotID, ix[1])
      }
~~~

```
   }

ssmod =bigssa (EMG ~ GRP2*TIME,
          type=list(TIME="cub",GRP2 ="nom"),
          rparm=list(TIME =0.01,GRP2 =1),
          nknots=knotID,
          data = dat,
          skip.iter=T)
ssmod$info
```

## Get CCD for paired conditions for LBP_N vs CON

```{r}
time = unique(dat$TIME)
grp <- c("CON","LBP_NO", "LBP_YES")

newdata =  expand.grid(TIME = time, GRP2 = grp)

yhat = predict(ssmod, newdata = newdata, se.fit=T,  design = T)

ccd_fit = newdata
ccd_fit$emg = yhat$fit
ccd_fit$X = yhat$X

con = ccd_fit[ccd_fit$GRP2 == "CON",]
lbp_n = ccd_fit[ccd_fit$GRP2 == "LBP_NO",]
lbp_y = ccd_fit[ccd_fit$GRP2 == "LBP_YES",]

colnames(con)[c(3,4)] = c("emg_con", "X_con")
colnames(lbp_n)[c(3,4)] = c("emg_lbp_n", "X_lbp_n")
colnames(lbp_y)[c(3,4)] = c("emg_lbp_y", "X_lbp_y")

ccd = lbp_n[c(3,4)]- con[c(3,4)]

const = con[,1:2]
ccd2 = cbind (const, ccd[,1] )
ccd2$X = as.matrix (ccd[,-1])


colnames (ccd2)[c(3:4)] = c("EMG", "X")
coefsqrt = ssmod $modelspec$coef.csqrt


### get Bayesian standard errors for CCDs
```

```
ccd2$se = sqrt(rowSums(((ccd2$X) %*% coefsqrt)^2))

ccd.plot = ccd2 %>% select (-X) %>%
  mutate (Comparison = "LBP_No vs Con")
```

## Get CCD for paired conditions for LBP_Y vs CON

```{r}
ccd = lbp_y[c(3,4)]- con[c(3,4)]

const = con[,1:2]
ccd2 = cbind (const, ccd[,1] )
ccd2$X = as.matrix (ccd[,-1])


colnames (ccd2)[c(3:4)] = c("EMG", "X")
coefsqrt = ssmod $modelspec$coef.csqrt


### get Bayesian standard errors for CCDs
ccd2$se = sqrt(rowSums(((ccd2$X) %*% coefsqrt)^2))

ccd.plot = ccd.plot %>%
  bind_rows(ccd2[,-4] %>% mutate (Comparison = "LBP_Yes vs Con"))

```

## Get CCD for paired conditions for LBP_N vs LBP_Y

```{r}
ccd = lbp_n[c(3,4)]- lbp_y[c(3,4)]

const = lbp_n[,1:2]
ccd2 = cbind (const, ccd[,1] )
ccd2$X = as.matrix (ccd[,-1])


colnames (ccd2)[c(3:4)] = c("EMG", "X")
coefsqrt = ssmod $modelspec$coef.csqrt


### get Bayesian standard errors for CCDs
ccd2$se = sqrt(rowSums(((ccd2$X) %*% coefsqrt)^2))

ccd.plot = ccd.plot %>%
  bind_rows(ccd2[,-4] %>% mutate (Comparison = "LBP_No vs LBP_Yes"))
```

```

## Plots for LBP_Y minus Con
```{r}

level = 0.95
cval = qnorm(1 - (1 - level)/2)


  ggplot(data= ccd.plot,
             aes(x = TIME, y = EMG)) +
    geom_line( aes (colour = as.factor (Comparison)), size = 1.5) +
    geom_ribbon(aes(ymin=EMG- cval*se, ymax=EMG + cval*se, fill = as.factor (Comparison)), 
alpha =0.2)  +
      scale_colour_manual(values = c("darkgreen", "red", "blue"),
                 name = "Group",
                 labels = c("LBP w/o FRP vs Control", "LBP w/o FRP vs LBP with FRP", "LBP 
with FRP vs control")) +
    scale_fill_manual(values = c("darkgreen", "red", "blue")) +
    guides(fill = F) +
    theme_bw() +
    labs (
      title = "Figure",
      x = "Time (s)",
      y =  "% EMG amplitude") +
    theme(panel.grid.minor = element_blank(),
         axis.line = element_line(colour = "black")) +
    geom_hline(yintercept=0)
